# Comparison of a General and Conditional Measure of E-Cigarette Harm Perceptions

**DOI:** 10.3390/ijerph17145151

**Published:** 2020-07-17

**Authors:** Olivia A. Wackowski, Michelle Jeong

**Affiliations:** Center for Tobacco Studies, Rutgers Biomedical and Health Sciences, New Brunswick, NJ 08901, USA; michelle.jeong@rutgers.edu

**Keywords:** harm perception, risk perception, e-cigarettes, measurement

## Abstract

Measures of tobacco product harm perceptions are important in research, given their association with tobacco use. Despite recommendations to use more specific harm and risk perception measures, limited research exists comparing different wordings. We present exploratory survey data comparing young adults’ (ages 18–29) responses to a general e-cigarette harm perception measure (“How harmful, if at all, do you think vaping/using an e-cigarette is to a user’s health?”) with a more specific conditional measure, which personalized the behavior/harm (“imagine you vaped,” “your health”) and presented a specific use condition (exclusive daily vaping) and timeframe (10 years). Data were collected in January 2019 (n = 1006). Measures were highly correlated (r = 0.76, Cronbach’s α = 0.86), and most (65%) provided consistent responses, although more participants rated e-cigarettes as very or extremely harmful using the conditional (51.6%) versus the general (43.9%) harm measure. However, significant differences in harm ratings were not observed among young adults who currently vaped. Correlations between each harm perception measure and measures of e-cigarette use intentions were similar. More specifically worded harm perception measures may result in somewhat higher e-cigarette harm ratings than general measures for some young adults. Additional research on best practices for measuring e-cigarette and other tobacco harm perceptions is warranted.

## 1. Introduction

Tobacco and nicotine product risk and harm perceptions have been associated with tobacco use, and as such, tobacco risk measures are important and frequently included in research studies [1,2,3]. However, these measures are often asked in a general way (e.g., “how harmful do you think e-cigarettes are to health?”) [1,4].

Recent reports and reviews of risk perception studies have recommended the use of more specific wording when measuring tobacco risk and harm perceptions [1,4,5]. This includes, for example, the measurement of harm/risk beliefs about specific health effects of a given product, rather than a single general harm or risk perception question [1,5,6,7,8,9]. Other recommendations include specifying for whom the risk is being evaluated (i.e., self or others), providing a conditional scenario of use (e.g., specifying the product used and level of exposure or frequency of product use), and providing a timeframe for consideration [1,4]. Providing this type of conditional detail may help to improve the likelihood that respondents are interpreting the question and risk assessment in a similar way, and may also aid in interpretation of responses [1]. However, a recent review of tobacco risk perception studies found that few included conditional measures that specified a frequency of product use and a timeframe, and that limited research exists comparing the results of conditional and unconditional tobacco risk measures [1]. A follow-up review also recommended more research to examine the potential advantage of conditional tobacco measures over unconditional measures and suggested that studies include both types of measures [4].

As such, this study presents exploratory data comparing responses to a general (unconditional) and more specific (conditional) measure of e-cigarette harm perceptions. The focus on e-cigarettes is important given the large growth in their use and their public health importance with respect to their potential use by smokers for harm reduction and their role in nicotine addiction and uptake in young people [10].

## 2. Materials and Methods

### 2.1. Participants

These pilot data were collected as part of a larger experimental study conducted in January 2019 about pictorial e-cigarette warning labels. Participants included 1006 young adult (ages 18–29) smokers and non-smokers in the USA, recruited through TurkPrime’s Prime Panels, which recruits participants from crowdsourcing platforms, such as Mechanical Turk (MTurk) as well as panels from other market research companies [11]. Participants were incentivized according to their respective survey panel from which they were recruited by TurkPrime.

### 2.2. Procedures

In the overall study, participants were randomly assigned to one of five conditions where they viewed two e-cigarette ads with a nicotine addiction warning label. Conditions varied by concept pictorial images included in the warning (text-only or one of four different images). After viewing the stimuli, participants responded to a series of outcome measures including intentions to use and buy e-cigarettes, as well as two questions about e-cigarette harm perceptions, the focus of this analysis.

### 2.3. Measures

*Harm perception measures.* All participants responded to two measures assessing e-cigarette harm perceptions: a general harm perception measure (“How harmful, if at all, do you think vaping/using an e-cigarette is to a user’s health?”) adapted from general measures of e-cigarette harm on large national surveys in the United States [12,13], and a conditional harm perception measure (“Imagine you vaped/used e-cigarettes daily for the next 10 years and used no other tobacco product. How harmful do you think this vaping would be to your health?”). The latter personalized the behavior and harm to the self (“imagine *you* vaped”; “*your* health”) and presented a specific use condition and timeframe (“daily for the next 10 years; “used no other tobacco product”), following recommendations and measures from previous studies [1,4,6,8]. In the current study, these two questions were presented to participants on separate online survey pages. Both measures used the same five-point response scale (not at all-extremely harmful).

*E-cigarette intentions.* We examined the association between participants’ e-cigarette harm perceptions and three different e-cigarette use intention measures: (1) interest in e-cigarette use (“How interested are you in vaping/using an e-cigarette in the next 6 months?”), (2) likelihood of e-cigarette use (“How likely are you to vape/use an e-cigarette in the next 6 months?”), and (3) likelihood of e-cigarette purchase (“How likely are you to buy an e-cigarette/vaping device in the next 6 months?”). All measures used a five-point response scale (not at all-extremely).

### 2.4. Analysis

For this exploratory study, we compared participants’ responses to both harm perception measures (general and conditional) in two ways: (1) treating them as numeric variables (examining inter-item correlation, internal consistency and mean responses using paired *t*-tests) and (2) as categorical variables, comparing the frequency of verbal response categories. For these categorical analyses, we dichotomized responses to the two e-cigarette harm measures as “very or extremely harmful” versus “not at all harmful, not very harmful or somewhat harmful” for ease of interpretation. We also compared categorical response consistency by coding provided responses that were the same to both measures as “consistent.” We coded the remaining cases as either “higher harm rating” or “lower harm rating” depending on whether the respondent gave the conditional item a higher or lower rating than the general measure.

In addition, we examined the associations between both harm perception measures and our three e-cigarette use intention measures with Pearson’s correlation tests. Based on previous research with young adults finding e-cigarette harm perception differences by user groups [14], current study results are presented for participants overall and by four mutually exclusive user categories—nonusers (who neither smoke nor vape), current exclusive cigarette smokers (i.e., currently smoke cigarettes “some days” or “every day” but do not currently use e-cigarettes), current exclusive e-cigarette users (i.e., currently vape “some days” or “every day” but do not currently smoke cigarettes), and dual users (i.e., participants who report both smoking and vaping “some days” or “every day”).

## 3. Results

The average age of participants was 24 (SD = 3), and 39% had at least a college degree. The majority of the sample was female (58%) and non-Hispanic white (59%). About 30% of participants both smoked cigarettes and used e-cigarettes currently (i.e., were “dual users”), while 12.3% only smoked cigarettes (i.e., “exclusive cigarette users”) and 8.4% only used e-cigarettes (i.e., “exclusive e-cigarette users”). Almost half (49.2%) did not currently use either product (i.e., “non-users”) (see Table 1).

*Item associations and consistency*. There was a significant positive correlation between the general harm measure and the conditional harm measure (r = 0.76, *p* < 0.001). When scaled together, Cronbach’s alpha was high (α = 0.86). In terms of response consistency, agreement on the categorical responses between the two measures was fair (Cohen’s kappa = 0.51, *p* < 0.001) [15]. While most participants (65%) provided the same verbal harm rating to both measures, 23% provided a higher harm rating to the conditional measure and 12% provided a lower harm rating to the conditional measure.

*Comparison of item responses*. Overall, more participants rated e-cigarettes as very or extremely harmful when presented with the conditional measure (51.6%) compared to when they were presented with the general measure (43.9%) (Table 1). Mean differences between the two measures were significantly different (conditional measure: 3.54 vs. general measure: 3.43; *p <* 0.001). When examining results by tobacco use status, mean harm perception ratings were significantly higher for the conditional versus the general measure among exclusive cigarette smokers and non-users, but did not differ by measure type among those who already used e-cigarettes (i.e., exclusive e-cigarette users and dual users) (Table 1).

*Association with use intentions*. Both harm measures (general and conditional) were negatively associated with future e-cigarette use intentions, such that those with higher e-cigarette harm perceptions had lower intentions to use or buy e-cigarettes in the next six months (Table 2). The strength of the association between e-cigarette harm perception measures and use intentions was highest among exclusive smokers. When comparing the strength of the associations by measure type (general and conditional measures), there were no significant differences for any of the three use intention outcomes (Table 2).

## 4. Discussion

Measurement of e-cigarette risk and harm perceptions is important given their association with product use [1,3,16,17]. Ideally, studies should use multiple items to increase the validity of perception measurement and researchers have also recommended research comparing risk/harm measures with more specific conditional wording to those with more general wording. In this exploratory survey study with young adults, we found high levels of correlation and internal consistency between participants’ responses to e-cigarette harm perception measures that used general and conditional wording, suggesting these types of measures may work well together on assessments.

Our results also suggest that, while these measures are strongly correlated, some young adults may trend towards providing higher e-cigarette harm ratings to conditional measures that provide more specific conditions of use (including use by oneself) than more generally worded measures. In our study this was observed in particular for young adults who did not already currently use e-cigarettes. It is perhaps unsurprising that those who were already using e-cigarettes, either exclusively or with cigarettes, seemed to be unaffected by the addition of conditional wording that presents a different scenario from their current behaviors. As current product users, these respondents may have already been thinking about themselves when answering the general version of the question, such that personalizing it further in the conditional measure did not make a substantive difference. Findings could also be related in part to the lowering and rationalization of tobacco product harms associated with trying to reduce feelings of cognitive dissonance that may occur after engaging in tobacco product use [16,17]. In this case, when motivated to reduce perceptions of harm, people may provide lower harm perceptions regardless of the specificity of the harm perception measures. Whereas the purpose of providing specific conditions is partly to ensure consistency of interpretation of the measure across participants, our findings suggest interpretation may differ somewhat depending on current tobacco use behaviors.

We also found that, while there were some differences in harm perception ratings by measure types, mean differences were fairly small, as indicated by the small effect sizes. Furthermore, there were no significant differences in how strongly these two measures correlated with e-cigarette use intentions, although it should be noted that use intentions may not always be predictive of actual product use.

Study limitations include the use of an online convenience sample. However, studies have found that, when examining non-experimental and experimental associations in the context of tobacco research, online convenience samples and national probability samples offer mostly consistent findings [18]. Although our sample size was large overall, the sample size of exclusive e-cigarette users (n = 85) was considerably smaller than other groups. In addition, these data were collected as part of a broader experimental study that was not specifically designed to test differences between types of e-cigarette harm perceptions, and as such, these data are exploratory. The two harm measures were shown in the same order for all participants (general followed by conditional measure), and thus, the study results may have been influenced to some extent by order effects, although this may have been mitigated by displaying these items on separate screens. Still, future studies aiming to build on this research would benefit from rotating the order of measures. Neither measure included a “don’t know” option, and it is unknown if such a response may have differed between the two measure types. This study only examined one conditional versus general measure comparison and did not examine the wording of measures focused on specific e-cigarette health effects. Furthermore, the measures we examined were of absolute harm from e-cigarettes rather than relative harm compared to cigarettes. Future research may benefit from additional study of the wording of relative risk/relative harm measures given that some work suggests these measures may better predict e-cigarette use than absolute harm/risk measures [3].

## 5. Conclusions

The results of more specifically worded e-cigarette harm perception measures may result in some different and potentially higher harm estimates among some young adults than more generally worded harm perception measures, although these measures are likely to be highly correlated and be similarly associated with e-cigarette use intentions. Additional research on best practices for tobacco risk perceptions measures, including those pertaining to e-cigarettes is warranted.

## Figures and Tables

**Table 1:** Comparison of e-cigarette harm perception responses by measurement type.

		Harm Measure Type ^a^		Harm Measure Type ^b^			
		General	Conditional	General	Conditional		
	N	% ^†^	% ^†^	Mean (SD)	Mean (SD)	(*p*)	Cohen’s d
All respondents	1006	43.9	51.6	3.43 (0.95)	3.54 (1.02)	<0.001	0.16
Nonusers	495	61.0	69.3	3.77 (0.88)	3.91 (0.88)	<0.001	0.21
Exclusive smokers	124	32.3	42.7	3.22 (0.93)	3.38 (1.01)	0.01	0.23
Exclusive e-cig users	85	23.5	35.3	3.11 (0.71)	3.28 (0.92)	0.15	0.27
Dual users	302	26.5	30.8	3.05 (0.94)	3.07 (1.03)	0.68	0.03

^a^ Responses analyzed as categorical variables; ^b^ Responses analyzed as numeric variables; ^†^ % of N responding “very or extremely harmful.”

**Table 2:** Associations between e-cigarette harm perceptions and e-cigarette intentions.

	Interest in Using E-cigs			Likelihood of Using E-cigs			Likelihood of Buying E-cigs		
	Gen	Con	*p* ^†^	Gen	Con	*p* ^†^	Gen	Con	*p* ^†^
All respondents	−0.418 **	−0.438 **	0.30	−0.415 **	−0.432 **	0.38	−0.381 **	−0.417 **	0.07
Nonusers	−0.239 **	−0.246 **	0.83	−0.258 **	−0.266 **	0.81	−0.196 **	−0.228 **	0.34
Exclusive smokers	−0.466 **	−0.423 **	0.45	−0.368 **	−0.282 *	0.15	−0.413 **	−0.348 **	0.27
Exclusive e-cig users	−0.244 **	−0.257 **	0.87	−0.251 **	−0.268 **	0.83	−0.185 **	−0.242 **	0.47
Dual users	−0.235 **	−0.211 **	0.55	−0.254 **	−0.234 **	0.61	−0.184 *	−0.211 **	0.50

Gen = general harm perception measure, Con = conditional harm perception measure. * *p* < 0.01; ** *p* < 0.001; asterisks indicate significance levels for Pearson’s correlations between each harm perception measure and the intention outcome; ^†^ *p* indicate significance levels for differences between correlation coefficients.
